# Severe Acute Respiratory Syndrome Coronavirus 2 ORF8 Protein Inhibits Type I Interferon Production by Targeting HSP90B1 Signaling

**DOI:** 10.3389/fcimb.2022.899546

**Published:** 2022-05-23

**Authors:** Jiayi Chen, Zixin Lu, Xiuwen Yang, Yezhen Zhou, Jing Gao, Shihao Zhang, Shan Huang, Jintai Cai, Jianhai Yu, Wei Zhao, Bao Zhang

**Affiliations:** ^1^BSL-3 Laboratory (Guangdong), Guangdong Provincial Key Laboratory of Tropical Disease Research, School of Public Health, Southern Medical University, Guangzhou, China; ^2^Department of Epidemiology, School of Public Health, Southern Medical University, Guangzhou, China

**Keywords:** SARS-CoV-2, ORF8 protein, type I interferon, HSP90B1, IRF3

## Abstract

Coronavirus disease 2019 (COVID-19), caused by severe acute respiratory syndrome coronavirus 2 (SARS-CoV-2), is a global pandemic that has currently infected over 430 million individuals worldwide. With the variant strains of SARS-CoV-2 emerging, a region of high mutation rates in ORF8 was identified during the early pandemic, which resulted in a mutation from leucine (L) to serine (S) at amino acid 84. A typical feature of ORF8 is the immune evasion by suppressing interferon response; however, the mechanisms by which the two variants of ORF8 antagonize the type I interferon (IFN-I) pathway have not yet been clearly investigated. Here, we reported that SARS-CoV-2 ORF8L and ORF8S with no difference inhibit the production of IFN-β, MDA5, RIG-I, ISG15, ISG56, IRF3, and other IFN-related genes induced by poly(I:C). In addition, both ORF8L and ORF8S proteins were found to suppress the nuclear translocation of IRF3. Mechanistically, the SARS-CoV-2 ORF8 protein interacts with HSP90B1, which was later investigated to induce the production of IFN-β and IRF3. Taken together, these results indicate that SARS-CoV-2 ORF8 antagonizes the RIG-I/MDA-5 signaling pathway by targeting HSP90B1, which subsequently exhibits an inhibitory effect on the production of IFN-I. These functions appeared not to be influenced by the genotypes of ORF8L and ORF8S. Our study provides an explanation for the antiviral immune suppression of SARS-CoV-2 and suggests implications for the pathogenic mechanism and treatment of COVID-19.

## Introduction

The outbreak of coronavirus disease 2019 (COVID-19) started in Wuhan, China, in December 2019, and the first cluster of COVID-19 cases was reported in the Huanan Seafood Wholesale Market (Huang et al., 2020). The pandemic triggered a series of cases of “unexplained pneumonia”, which was identified to be caused by a new virus that is now officially named severe acute respiratory syndrome coronavirus 2 (SARS-CoV-2) (Coronaviridae Study Group of the International Committee on Taxonomy of Viruses, 2020; Zhou et al., 2020). The sequence of SARS-CoV-2 has approximately 80% similarity with that of SARS-CoV, but it is relatively distinct from the Middle East respiratory syndrome coronavirus (MERS-CoV), with only ~50% identity genomically (Chan et al., 2020; Lu et al., 2020). SARS-CoV-2, like SARS-CoV and MERS-CoV, belongs to the Coronaviridae family and is classified as a β-CoV (Betacoronavirus genus). It is an enveloped virus with single-stranded, positive-sense RNA of approximately 29.9 kb in size and contains 11 open reading frames (ORFs) (Wu et al., 2020). The genomic RNA encodes viral structural proteins, including the spike (S), membrane (M), envelope (E), and nucleocapsid (N), non-structural proteins, and accessory proteins. These accessory proteins, such as ORF3, 6, 7, 8, and 10, play an essential role in viral replication, viral release, virus pathogenesis, and immune evasion (Yadav et al., 2021). Compared to SARS-CoV and MERS-CoV, SARS-CoV-2 has evolved the capability to infect and transmit among the hosts, which has currently infected over 430 million individuals and caused nearly 5.9 million deaths worldwide (till February 2022), according to the WHO COVID-19 Dashboard (https://covid19.who.int/). Previous epidemiological studies have reported that a substantial proportion of infected individuals were asymptomatic, which may be associated with viral immune evasion. However, a distinct phenotype was observed in severe and critical patients, consisting of highly impaired type I interferon (IFN-I) responses (characterized by no IFN-β and low IFN-α production and activity), which correlated with a persistent blood viral load and an exacerbated inflammatory response. These data suggest that IFN-I deficiency in the blood could be a hallmark of severe COVID-19, and thus the virus could not be effectively cleared by the immune system and progresses to a critical stage (Hadjadj et al., 2020).

The IFN-I response is essential for effective resistance to viral infection and efficient protection of the organism. The recognition of pathogen-associated molecular patterns (PAMPs) by host cells rapidly triggers IFN-I production, which is mediated by transcription factors that induce the expression of interferon-stimulated genes (ISGs) (Schoggins, 2019). ISGs and pro-inflammatory cytokines regulated by IFN-I directly inhibit viral replication and recruit various immune cells to facilitate viral clearance (Crouse et al., 2015; Makris et al., 2017). As with most RNA viruses, coronavirus RNA is recognized by retinoic acid-inducible gene I (RIG-I)/melanoma differentiation-associated gene 5 (MDA5) (Li et al., 2010), which interact with mitochondrial antiviral signaling (MAVS) to initiate antiviral signaling and then activate TRAF family member-associated NF-κB Activator (TANK) binding kinase 1 (TBK1) and inhibitor of nuclear factor κB (IκB) kinase ε (IKKϵ). Phosphorylation and dimerization of the IFN regulatory factors (IRF3 and IRF7) are translocated to the nucleus, which induces the expression of IFN-I and ISGs (Loo and Gale, 2011). The secreted IFN-I binds specific interferon receptors, triggering the activation of Jak tyrosine kinase 2 (Tyk2) and Janus kinase 1 (JAK1), which stimulate the phosphorylation of signal transducer and activator of transcription (STAT1 and STAT2). Subsequently, p-STAT1 and p-STAT2 associate with IRF9 to form the IFN-stimulated gene factor 3 (ISGF3) complex, which is translocated into the nucleus and binds IFN-stimulated response element (ISREs) in the ISG promoter, thereby inducing the expression of ISGs with antiviral ability (Schindler et al., 2007).

Currently, it is well-established that dozens of viral proteins encoded by the SARS-CoV or MERS-CoV genome are associated with virus escape and diminishment of IFN induction. Although the SARS-CoV-2 genome shares ~80% identity with SARS-CoV, a better understanding of the mechanism is required to determine whether specific viral proteins of SARS-CoV-2 might also exhibit an IFN-antagonizing activity. Studies have shown that innate immune signaling proteins are screened for interaction with SARS-CoV-2 genomic proteins by mass spectrometry; however, no relevant experiments have been performed to verify the interactions (Gordon et al., 2020). Co-immunoprecipitation (co-IP) and immunofluorescence (IF) assays demonstrate that SARS-CoV-2 ORF9b, like SARS-CoV ORF9b, is localized in mitochondria and inhibits IFN-I response by interacting with TOM70 (Jiang et al., 2020). Furthermore, SARS-CoV ORF9b causes mitochondrial elongation by triggering ubiquitination and protease degradation of dynamin-like protein (DRP1) and subsequently results in degradation of MAVS, TRAF3, and TRAF6 by usurping poly(C)-binding protein 2 (PCBP2) and the HECT structural domain E3 ligase AIP4, thereby inhibiting the interferon response in host cells (Shi et al., 2014). By analyzing 17,000 SARS-CoV-2 sequences, a natural variant that encodes longer SARS-CoV-2 ORF3b was determined to exhibit the ability of suppressing interferon induction more efficiently (Konno et al., 2020). SARS-CoV-2 NSP13, NSP14, NSP15, and ORF6 could potently inhibit primary interferon production and interferon signaling; however, PL^pro^ loses interferon antagonism and deubiquitinase activity as well as fails to suppress interferon production (Yuen et al., 2020). Taken together, structural, non-structural, or accessory proteins of SARS-CoV-2 are potential IFN-I antagonists and therefore exhibit an inhibitory ability *via* interacting with different factors in the IFN-I signaling pathway.

Furthermore, the SARS-CoV-2 genome shows high variability at two core positions, one is a silent variant in the ORF1ab locus, and the other is an amino acid polymorphism in ORF8. The mutation of ORF8 at amino acid 84 results in two variants, ORF8L (leucine) and ORF8S (serine), while the remaining gene loci are completely concordant to both (Chan et al., 2020; Mohammad et al., 2020). It is predicted that the substitution of the non-polar amino acids (leucine, L) for the polar ones (serine, S) may affect the conformation of both polypeptides, which, in turn, may lead to differences in protein structure, but there is little experimental support for these hypotheses at present. The SARS-CoV-2 ORF8 protein is far from homologous to the SARS-CoV ORF8 protein. The sequence homology between SARS-CoV-2 and SARS-CoV from early-phase patients or animals in 2003, both of which contain a full-length ORF8, was ~26%, while the deletion of 29 nucleotides results in splitting ORF8 into ORF8a and ORF8b, which were present in mid- and late-phase patients. The SARS-CoV-2 ORF8 protein was more distantly related to ORF8a (10% identity) and ORF8b (16% identity) of SARS-CoV (SARS-CoV_BJ01) (Zhang et al., 2021). Several studies have suggested that ORF8 acts as IFN-I antagonists, in both SARS-CoV and SARS-CoV-2. ORF8b and ORF8ab proteins in SARS-CoV effectively inhibited IFN-β signaling pathway by interacting with IRF3, which was partially mediated by protein 8b/8ab-induced degradation of IRF3 in a ubiquitin-proteasome-dependent manner (Wong et al., 2018). In SARS-CoV-2, the ORF8 protein interacts with major histocompatibility complex class I (MHC-I) molecules that are selectively targeted for lysosomal degradation *via* autophagy, and thus, SARS-CoV-2-infected cells are much less sensitive to lysis by cytotoxic T lymphocytes and in turn evade immune surveillance (Zhang et al., 2021). On the other hand, the ORF8 protein was found to be a potential inhibitor of the IFN-I signaling pathway, which showed strong suppression on IFN-β and the NF-κB-responsive promoter, and even the interferon-stimulated response element (ISRE) after infection with the Sendai virus (Li et al., 2020). Moreover, both ORF8L and ORF8S proteins could induce endoplasmic reticulum (ER) stress by triggering the activating transcription factor 6 (ATF6) and inositol-requiring enzyme 1 (IRE1) pathways, and thus inhibit IFN-β production as well as decrease nuclear translocation of IRF3 (Rashid et al., 2021). Moreover, the host immune system could be rescued by novel drugs *via* targeting the immune evasion complex ORF8–IRF3 in SARS-CoV-2 infection using molecular modeling approaches (Albutti, 2021).

SARS-CoV-2 ORF8 may not interact with IRF3 directly; however, the interaction between HSP90 and IRF3 activated IRF3 and stabilized TBK1 in Sendai virus-infected cells (Yang et al., 2006). Several studies have demonstrated that HSP90 is associated with virus infection. Inhibition of HSP90 activity reduced SARS-CoV-2 replication and pro-inflammatory cytokine expression in human respiratory epithelial cells (Wyler et al., 2021). The RNA synthesis and subsequent virus production of the Porcine Reproductive and Respiratory Syndrome virus (PRRSV) were reduced *via* suppressing the expression of HSP90 (Gao et al., 2014). Furthermore, the interaction of SARS-CoV-2 ORF9b with TOM70 inhibited the recruitment of HSP90 and chaperone proteins to reduce the expression of IFN-I (Brandherm et al., 2021). Moreover, a system biology analysis of the protein–protein interaction (PPI) with SARS-CoV-2 indicated ten hub proteins, including HSP90B1, which may be therapeutic targets in COVID-19 patients with CKD (chronic kidney disease) comorbidity (Auwul et al., 2021). Therefore, the interactions between SARS-CoV-2 ORF8 and HSP90B1, associated with IRF3 in IFN-I pathways, merit extensive investigation.

Overall, under most circumstances, IFN-I pathways play an essential role in preventing infection and replication of SARS-CoV-2. For its defense, multiple viral proteins encoded by SARS-CoV-2 antagonize IFN response to resist immune clearance and progress into severe and critical diseases. Nevertheless, the association between the infection of SARS-CoV-2 and host antiviral immunity is not fully understood yet. Thus, the mechanism by which viral proteins assist SARS-CoV-2 to evade the IFN-I response needs investigation and clarification. Here, we reported that SARS-CoV-2 ORF8 acts as an antagonist of IFN-I response by targeting HSP90B1, in both ORF8L and ORF8S genotypes with no difference. ORF8 inhibited IFN-I production and RIG-I like receptor (RLR) pathway signaling molecules induced by poly(I:C) transfection, which was triggered by decreasing the nuclear translocation of IRF3. The interaction between ORF8 and HSP90B1 exerts inhibitory influence on IFN-I response and thus achieves immune escape. This study elucidated the mechanism by which SARS-CoV-2 ORF8 proteins inhibit host antiviral immunity, enlightening the understanding of viral pathogenesis of SARS-CoV-2 and targeted therapy of COVID-19.

## Materials and Methods

### Cell Culture and Transfection

Both HeLa and HEK293T cells were cultured in GIBCO Dulbecco’s modified Eagle medium (DMEM, Gibco, USA) supplemented with 10% fetal bovine serum (FBS, Gibco, USA) at 37°C in a humidified 5% CO_2_ atmosphere. Cells (2.5 × 10^5^ cells/well) were seeded in a 12-well plate or 100-mm dishes. After 24 h, plasmids, siRNA, or poly(I:C) (InvivoGen, USA) was transfected into HEK293T and HeLa cells with Lipofectamine 3000 (Thermo Fisher Scientific, USA) according to the manufacturer’s recommendations. The sequence of the siRNA specific for HSP90B1 was 5’-CTTCGCCTCAGTTTGAACA-3’. Both siRNA-HSP90B1 and siRNA-NC (5 nmol, siN0000001-1-5) were purchased from RiboBio (China).

### Construction of the Protein Expression Vectors

Full-length ORF8L and ORF8S were amplified and cloned into the corresponding restriction sites (*Bam*HI) of the plasmid pEGFP-N1 to generate the recombinant plasmids pEGFP-ORF8L and pEGFP-ORF8S, respectively. Site-directed mutagenesis was used to insert the Myc-tag into the pEGFP-ORF8L and pEGFP-ORF8S to generate pEGFP-ORF8L-Myc and pEGFP-ORF8S-Myc plasmids. The primers used for plasmid construction are listed in **Supplementary Table 1**. All recombinant plasmids were further verified by DNA sequencing and transfected into cells for Western blot.

### Cell Counting Kit-8 Assay

The HeLa cells were seeded into 24-well plates (1×10^5^ per well) with 500 μl of culture medium and transfected with pEGFP-N1, pEGFP-ORF8L, and pEGFP-ORF8S for 24 h or 48 h. CCK-8 solution (Fdbio science, China) was added into the wells (30 μl per well) followed by incubation at 37°C for 2 h and then spectrophotometric data were measured using a spectrophotometer system (Infinite M200, Tecan, Switzerland) at a wavelength of 450 nm.

### RT-PCR

Total RNA was isolated with TRIzol Reagent (Accurate Biology, China) and then reverse transcribed to first-strand cDNA with the Evo M-MLV RT Kit with gDNA Clean (Accurate Biology, China) according to the manufacturer’s instructions. Real-time quantitative PCR (RT-PCR) assays were performed by SYBR^®^ Green Premix Pro Taq HS qPCR Kit (Accurate Biology, China) in a Roche LightCycler 96 system. The relative abundances of the indicated mRNA transcripts were normalized to those of GAPDH and were expressed as fold changes over control calculated by the 2^−ΔΔCt^ method. All primers used for RT-qPCR analysis are provided in **Supplementary Table 2**.

### Western Blot Analysis

Cells were lysed in RIPA buffer (Beyotime, China) with protease inhibitor cocktail (Bimake, USA) and PMSF (GBCBIO Technologies, China) on ice for 30 min. After the supernatant was harvested and total protein concentration was determined by a BCA protein assay kit (Bioworld, USA), protein lysates were separated by SDS-PAGE and transferred to polyvinylidene difluoride (PVDF) membranes (Merck, Germany) *via* semi-dry transfer (Bio-Rad, USA). The membranes were blocked with 5% non-fat milk in PBST for 2 h at room temperature before incubating with specific primary antibodies, followed by Goat anti-Rabbit IgG (H+L)-HRP or Goat anti-Mouse IgG (H+L)-HRP (Bioworld, USA), respectively. The following primary antibodies were used: β-actin, Myc-tag (Proteintech, China), EGFP (abcam, UK), and anti-HSP90B1 (Proteintech, China).

### Organelle Staining

HeLa cells were cultured in confocal dishes and transfected with indicated plasmids for 48 h. After the culture medium was discarded and the cells were washed three times with PBS, HeLa cells were stained with organelle-specific fluorescence dyes, such as ER-, Golgi-, Mito-, and Lyso-Tracker-Red (Beyotime, China), according to the manufacturer’s instructions. Nuclei were stained with Hoechst 33342 (Beyotime, China). Images were acquired with a Zeiss LSM880 confocal microscope (Germany) and analyzed with ZEN (blue version).

### Confocal Immunofluorescence Microscopy

HeLa cells or HEK293T cells grown on coverslips in 6-well plates were transfected with corresponding plasmids for 48 h. Cells were fixed with 4% paraformaldehyde, permeabilized with 0.5% Triton X-100, and blocked with phosphate-buffered saline (PBS) containing 10% goat serum and 0.1% Triton X-100. The cells were then incubated with the indicated primary antibodies at 4°C overnight and with goat anti-mouse-CoraLite 594 antibodies (Proteintech, China) for 1 h. Nuclei were stained with DAPI (Bestbio, China). The IRF3 antibodies were purchased from Proteintech (China). Images were acquired with a Zeiss LSM880 confocal microscope (Germany) and analyzed with ZEN (blue version) and ImageJ.

### Co-Immunoprecipitation Analysis

HEK293T cells grown overnight in 100-mm dishes were transfected with pEGFP-ORF8L-Myc and pEGFP-ORF8S-Myc using Lipofectamine 3000 (Thermo Fisher Scientific, USA). At 48 h post-transfection, cells were harvested with 500 μl of IP lysis buffer (Beyotime, China) in the presence of protease inhibitor cocktail (Bimake, USA) and PMSF (GBCBIO Technologies, China). Lysates were centrifuged at 15,000 × *g* on ice for 15 min, and then 1 mg of supernatant protein was incubated with 3 μg of the indicated antibodies overnight, followed by 40 μl of protein A+G agarose (Beyotime, China) for 5 h at 4°C with gentle shaking. The beads were washed three times with 500 μl of IP lysis buffer and eluted by boiling with 2 × SDS-PAGE loading buffer (CWBio, China), before the immunoprecipitated proteins were separated on SDS-PAGE and analyzed by Western blot using appropriate antibodies. The anti-Myc-tag and anti-HSP90B1 were purchased from Proteintech (China), while antibodies against rabbit IgG were from abclonal (China).

### Statistical Analysis

The results from three independent experiments are presented as mean ± SD values by GraphPad Prism 8. For statistical analysis, one-way ANOVA or Student’s *t*-test was performed in SPSS 25.0. Differences were considered statistically significant at a value of *p* < 0.05.

## Results

### The Subcellular Localization of SARS-CoV-2 ORF8L and ORF8S Proteins

**Figure 1:**
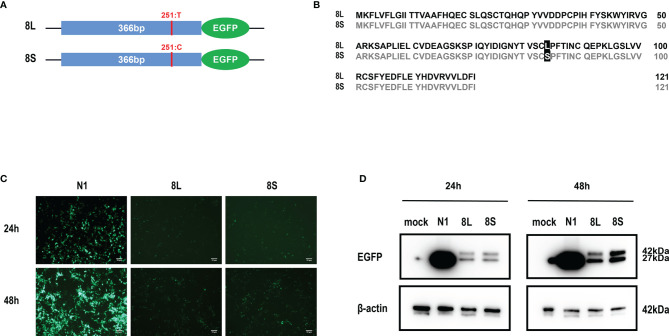
Expression of pEGFP-ORF8L and pEGFP-ORF8S in HEK293T cells. **(A)** Schematic diagram of ORF8L and ORF8S expression vectors. The only mutation of nucleotide 251 was T in ORF8L and C in ORF8S. **(B)** Alignment of amino acid sequence in ORF8L and ORF8S. Leucine in ORF8L was substituted by serine in ORF8S at amino acid 84. **(C)** Images of fluorescence-labeled ORF8L and ORF8S proteins in cells were obtained by fluorescence microscopy. Scale bar, 10 μm. **(D)** Expression of ORF8L and ORF8S proteins was examined by Western blot assay. The ORF8L-EGFP and ORF8S-EGFP proteins have a putative molecular weight of nearly 42 kDa, and EGFP protein alone has a molecular weight of 27 kDa. pEGFP-N1, N1; pEGFP-ORF8L, 8L; pEGFP-ORF8S, 8S.

**Figure 2:**
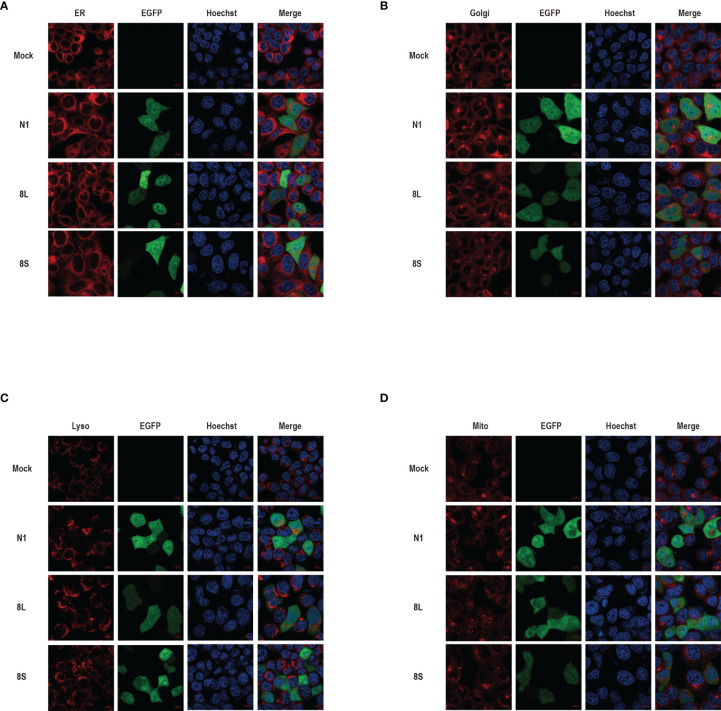
The cellular sub-localization of SARS-CoV-2 ORF8L, and ORF8S proteins. HeLa cells were transfected with pEGFP-N1, pEGFP-ORF8L, and pEGFP-ORF8S for 48 h; fixed; and then stained with Tracker-Red against endoplasmic reticulum **(A)**, Golgi apparatus **(B)**, lysosome **(C)**, and mitochondria **(D)** (red). ORF8L and ORF8S proteins were shown in green while nucleus was visualized with Hoechst (blue). Confocal imaging results are representative of two independent experiments. Scale bar, 10 μm. Endoplasmic reticulum, ER; Golgi apparatus, Golgi; lysosome, Lyso; mitochondria, Mito; pEGFP-N1, N1; pEGFP-ORF8L, 8L; pEGFP-ORF8S, 8S; hours, h.

We constructed pEGFP-N1 expression vector expressing ORF8L (251:T) and ORF8S (251:C) proteins. The EGFP-tag proteins were detected at the correct molecular weight, indicating that ORF8L and ORF8S fusion proteins were successfully expressed (**Figure 1**). SARS-CoV-2 ORF8 proteins have no significant impact on the viability of HeLa cells (shown in **Supplementary Figure 1**), indicating that ORF8 proteins did not show toxic effects to the cells. To determine the cellular location of ORF8L and ORF8S proteins, pEGFP-N1, pEGFP-ORF8L, and pEGFP-ORF8S were transfected in HeLa cells, and the corresponding organelles were labeled with red fluorescent probes for ER, Golgi apparatus, lysosomes, and mitochondria, respectively. The ORF8 protein was indicated by green fluorescence while the organelles were marked by red fluorescence, which were observed under a laser confocal fluorescence microscope, so as to explore the cellular sub-localization of ORF8L and ORF8S proteins. The results showed that both ORF8L and ORF8S proteins were uniformly distributed in the cytoplasm and nucleus of HeLa cells. A weaker distribution of ORF8L and ORF8S proteins could be observed in four organelles: ER, Golgi apparatus, lysosomes, and mitochondria (**Figures 2A–D**), which demonstrated that SARS-CoV-2 ORF8L and ORF8S were diffusely distributed throughout the cell and do not concentrate or reside in specific organelles.

### The SARS-CoV-2 ORF8L and ORF8S Inhibit Type I IFN Induction by Poly(I:C)

**Figure 3:**
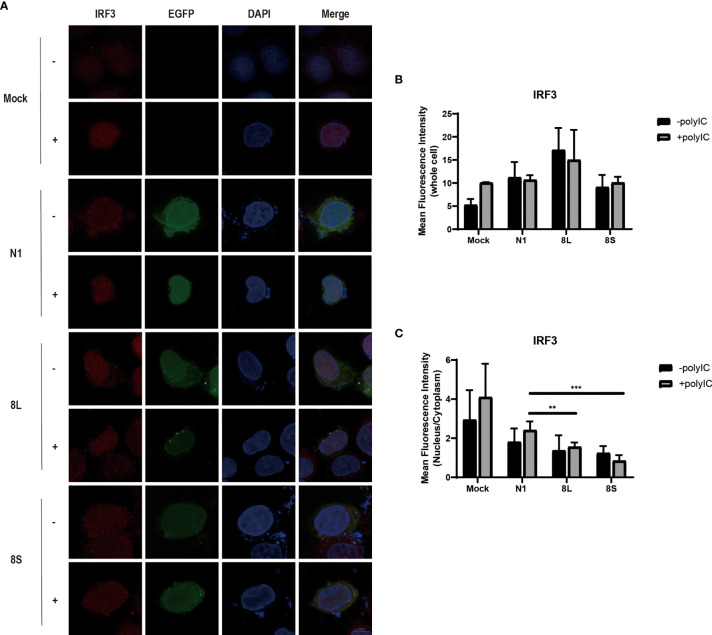
The SARS-CoV-2 ORF8L and ORF8S proteins inhibit nucleus translocation of IRF3. **(A)** HeLa cells were co-transfected with indicated plasmids and poly(I:C) [-, no poly(I:C); +, poly(I:C)]. At 48 h after transfection, the cells were fixed, blocked, and then incubated with a rabbit anti-IRF3 antibody overnight and with the corresponding coraLite594 antibody for 1 h and a confocal microscope was used to identify ORF8L, ORF8S proteins (green), and IRF3 (red) signals. The mean fluorescence intensity of IRF3 of whole cells **(B)** or nucleus to cytoplasm **(C)** in positive-transfected cells was measured from over three cells in three fields of views. Scale bar, 10 μm. **P < 0.01; ***P < 0.001 versus empty vector (One-way ANOVA). ORF8L protein, 8L; ORF8S protein, 8S.

**Figure 4:**
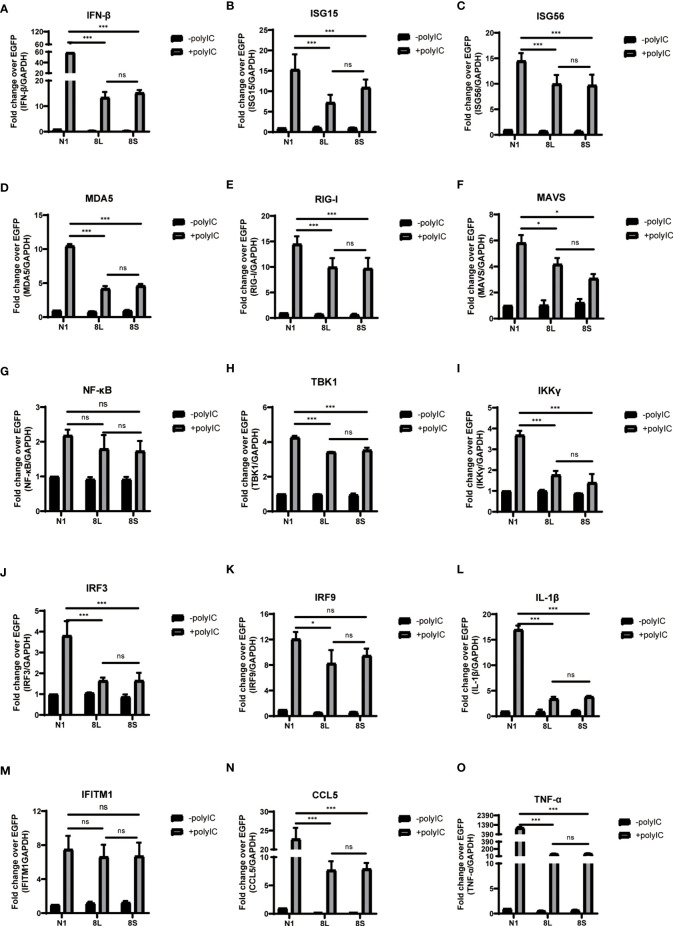
The ORF8L and ORF8S proteins suppress the induction of cytokines in RLR pathways by poly(I:C) transfection. HeLa cells cultured in 12-well plates were co-transfected with control vector pEGFP-N1, pEGFP-ORF8L, and pEGFP-ORF8S, respectively, together with poly(I:C) (1 μg/ml). At 24 h post-transfection, cells were lysed and total RNA was then extracted for quantitative real-time RT-PCR with specific primers for cytokines **(A–O)**. The expression of each gene was expressed relative to their respective control sample transfected with empty vector. ns, no significance; *P < 0.05; ***P < 0.001 versus empty vector (one-way ANOVA). Three independent biological replicates were analyzed, the results of one representative experiment are shown, and the error bars indicate the SD value. pEGFP-N1, N1; pEGFP-ORF8L, 8L; pEGFP-ORF8S, 8S.

To explore whether the SARS-CoV-2 ORF8L and ORF8S proteins affect type I IFN response, HeLa cells expressing SARS-CoV-2 ORF8 were transfected with dsRNA mimic poly(I:C), and the expression levels of IFN-β, MDA5, RIG-I, ISGs, etc. were measured by qPCR. Poly(I:C) transfection strongly stimulated the expression of IFN-β, MDA5, RIG-I, ISGs, etc. in control HeLa cells, leading to cytokine storms as SARS-CoV-2 did (Ye et al., 2020; Hu et al., 2021). In HeLa cells expressing the ORF8 protein, the induction of IFN-β, MDA5, RIG-I, ISGs, etc. was significantly inhibited compared with that in HeLa cells transfected with an empty vector (**Figure 4**). Furthermore, to determine the possible inhibitory mechanism of ORF8L and ORF8S in suppressing IFN induction, the nucleus translocation of IRF3 was detected after expressing ORF8L, ORF8S, and the empty vector in the presence or absence of poly(I:C). The results suggested that the nucleus translocation of IRF3 was significantly decreased by ORF8L and ORF8S compared with the empty vector (**Figure 3**). The genotypes of ORF8L and ORF8S were indistinguishable in all findings above.

### The SARS-CoV-2 ORF8L and ORF8S Proteins Interact With HSP90B1

**Figure 5:**
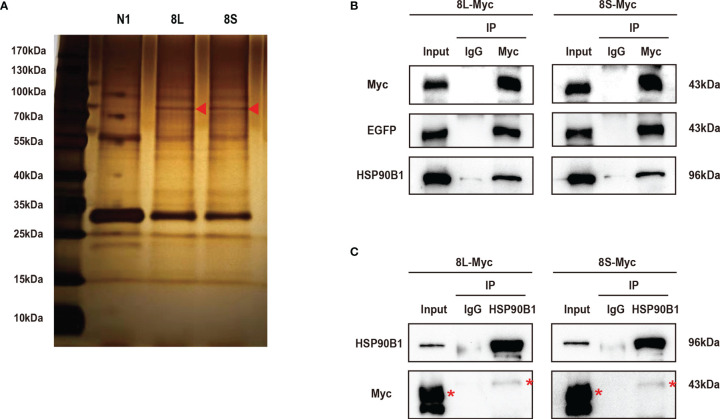
The SARS-CoV-2 ORF8L and ORF8S proteins interact with HSP90B1. **(A)** The HEK293T cells were transfected with pEGFP-ORF8L and pEGFP-ORF8S for 48 h. The cell lysates were immunoprecipitated with anti-GFP pAb and then were separated by SDS-PAGE, followed by silver staining and LC-MS/MS. Shown are the differential protein bands between EGFP-8L- or EGFP-8S- and EGFP-expressing cells. Red triangles indicate the band from which HSP90B1 was identified. **(B)** The HEK293T cells were transfected with pEGFP-ORF8L-Myc and pEGFP-ORF8S-Myc for 48 h. The cell lysates were co-immunoprecipitated with anti-Myc antibody and detected with anti-Myc, anti-EGFP, and anti-HSP90B1 antibody by Western blot. **(C)** The indicated plasmids were transfected into HEK293T cells before co-immunoprecipitation with anti-HSP90B1 antibody and were analyzed by Western blot with anti-Myc antibody. Red asterisks indicate the target band. Co-immunoprecipitation results are representative of two independent experiments. pEGFP-ORF8L-Myc, 8L-Myc; pEGFP-ORF8S-Myc, 8S-Myc.

To further study the mechanism by which the ORF8L and ORF8S proteins regulate IFN response, co-immunoprecipitation (Co-IP) experiments were performed to assess interactions between ORF8L, ORF8S, and RLR signaling molecules. We transfected pEGFP-N1, pEGFP-ORF8L, and pEGFP-ORF8S into HEK293T cells for 48 h and the cells were harvested and immunoprecipitated with anti-GFP antibody and then subjected to SDS-PAGE and visualized by silver staining (**Figure 5A**). Compared with the control lane, differential bands were excised and identified by LC-MS/MS. Of all these proteins that may interact with ORF8L and ORF8S, HSP90B1 was chosen for further analysis due to its higher score and its specific functions (**Supplementary Table 3**). To confirm the interaction between HSP90B1 and ORF8L and between HSP90B1 and ORF8S, plasmids pEGFP-ORF8L-Myc and pEGFP-ORF8S-Myc were constructed (shown in **Supplementary Figure 2**) and transfected into HEK293T. At 48 h post-transfection, the cell lysates were co-immunoprecipitated with anti-Myc antibody or with normal IgG as negative control and then subjected to Western blot. The results showed that HSP90B1 interacted with ORF8L and ORF8S in Co-IP with either anti-Myc pAb (**Figure 5B**) or anti-HSP90B1 pAb (**Figure 5C**).

### ORF8 Protein Suppresses Type I Interferon Response *via* HSP90B1

**Figure 6:**
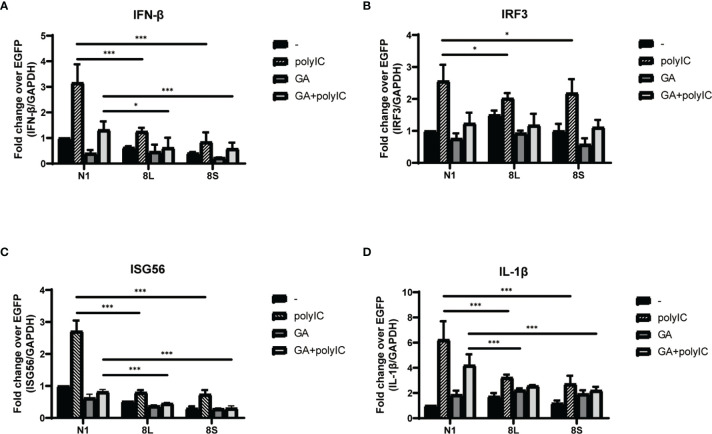
The SARS-CoV-2 ORF8L and ORF8S suppress IFN response with antagonism of HSP90. **(A–D)** HeLa cells were seeded in 12-well plates for 12 h and incubated with GA (1 μM) for the succeeding 12 h, after which the indicated plasmids were transfected into HeLa cells and cells were treated with GA (1 μM) at 6 h post-transfection. After 24 h of transfection, the mRNA of cytokines in IFN pathways was examined by RT-PCR. *, P < 0.05; ***P < 0.001 versus empty vector (one-way ANOVA). Three independent biological replicates were analyzed, the results of one representative experiment are shown, and the error bars indicate the SD value.

**Figure 7:**
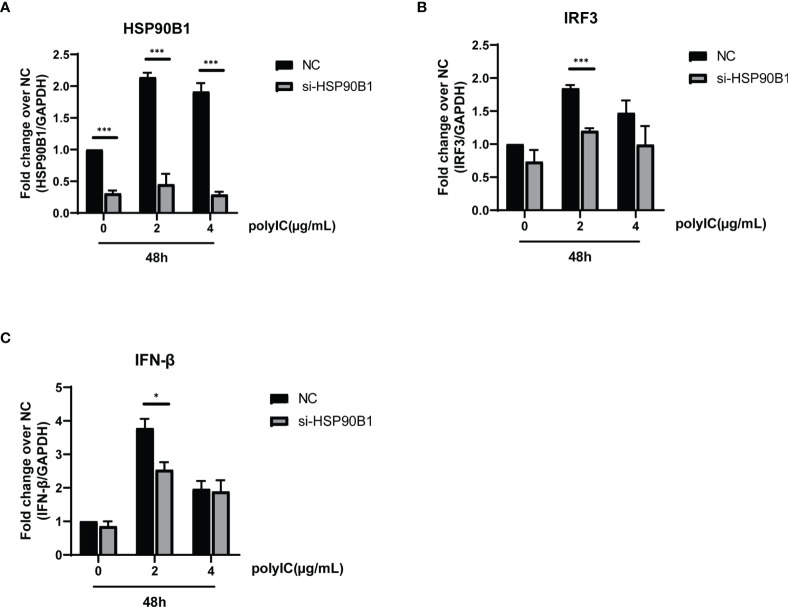
Knockdown of HSP90B1 inhibits IFN pathways induced by poly(I:C). **(A–C)** HeLa cells were plated in 12-well plates and co-transfected with si-HSP90B1 and poly(I:C) at the indicated concentration. At 24 h post-transfection, mRNA of HSP90B1 and cytokines in IFN pathways were measured by RT-PCR. *P < 0.05; ***P < 0.001 versus empty vector (one-way ANOVA). Three independent biological replicates were analyzed, the results of one representative experiment are shown, and the error bars indicate the SD value.

To explore the relationship between HSP90B1 and IFN response, Geldanamycin (GA), an HSP90 inhibitor, was used to suppress HSP90 expression. HeLa cells were treated with GA before and after co-transfection of indicated plasmids and poly(I:C). One study reported that in Sendai virus-infected cells, HSP90 interacted with IRF3 to activate IRF3 and stabilize TBK1 (Yang et al., 2006). We observed that GA is an antagonist of IFN pathways, suppressing the expression of IFN-β, ISG56, IRF3, and IL-1β. Moreover, ORF8L and ORF8S shared similar trends of inhibiting IFN-β, ISG56, and IL-1β with the treatment of GA, suggesting that HSP90 may play an important role in suppressing IFN pathways by ORF8L and ORF8S (**Figure 6**). To confirm whether HSP90B1, a member of HSP90 family (Marzec et al., 2012; Hoter et al., 2018), is associated with IFN response, siRNA targeting HSP90B1 was used to silence HSP90B1 and co-transfected into HeLa cells with poly(I:C). High knockdown efficiency (>80%) was achieved at the indicated concentration of poly(I:C), and IFN-β and IRF3 could be significantly downregulated by silencing HSP90B1, indicating that ORF8L and ORF8S may regulate the IFN pathways by interacting with HSP90B1, thereby inhibiting the expression of interferon and other IFN-related cytokines (**Figure 7**).

## Discussion

Previous studies have demonstrated that antiviral innate immunity plays an important role in viral elimination of SARS-CoV-2 in COVID-19 patients (Boechat et al., 2021). However, dysfunction of antiviral innate immunity and inflammatory responses mediated by SARS-CoV-2 is largely responsible for severe or critical disease and even death caused by COVID-19 (Hadjadj et al., 2020; Boechat et al., 2021). An unbalanced immune response characterized by weak or inhibitory production of IFN-I was observed in COVID-19; nevertheless, the pathogenic mechanism of IFN antagonism induced by SARS-CoV-2 is unclear and requires further elucidation. Here, we reported that SARS-CoV-2 ORF8L and ORF8S proteins inhibit IFN-I production by targeting HSP90B1 that is associated with IRF3, thus suppressing host antiviral immunity.

The research on sub-localization of viral genome proteins has a significant impact on the investigation of their function in the viral infection of organisms. In this experiment, ORF8 proteins were found to be diffusely distributed in both the cytoplasm and nucleus, and could weakly localize in the ER, Golgi apparatus, lysosome, and mitochondria, indicating that ORF8L and ORF8S proteins are not concentrated in the cytoplasm or reside in specific organelles, although some scholars believed that ORF8 is involved in the synthesis of disulfide bonds based on its cysteine residue, and thus speculated that ORF8 is an ER-resident protein. According to the co-IP analysis, ORF8 is also associated with the protein quality control process in ER (Mohammad et al., 2020). In addition, our studies proved that ORF8 interacts with HSP90B1, a molecular chaperone of ER, which also confirmed that ORF8 has a certain relationship with ER, but cannot explain whether ORF8 can reside in it. However, other studies suggested that ORF8 is a secreted protein, which is not localized in ER but concentrated near the cell surface (Wang et al., 2020). Therefore, the subcellular localization of ORF8, including whether it resides in the ER or other organelles, still needs to be verified by further experiments.

The proteins encoded by coronavirus genome have different viral evasion strategies against the IFN response, such as inhibiting IFN induction, suppressing IFN signaling, and increasing IFN resistance, thereby helping the virus to evade immune clearance. The ORF8b and ORF8ab proteins but not ORF8a proteins of SARS-CoV have been proven to suppress IFN-β signaling pathway by interacting with IRF3 to trigger its degradation in a ubiquitin–proteasome-dependent manner (Wong et al., 2018). However, MERS-CoV ORF8b proteins have not been reported to show a similar trend of IFN-I inhibition so far. Importantly, recent studies found that SARS-CoV-2 ORF8 proteins may function as IFN-I inhibitors by suppressing IFN-β- and NF-κB-responsive promoters and ISRE (Li et al., 2020). Furthermore, ORF8L and ORF8S proteins can induce ER stress to decrease IFN-β production as well as nuclear translocation of IRF3 (Rashid et al., 2021), while SARS-CoV-2 ORF8 activates CTP synthetase 1 (CTPS1) and thus induces the deamidation of IRF3, which fails to bind the promoters of classic IRF3-responsible genes, therefore muting IFN induction (Rao et al., 2021). However, the current studies have not fully elucidated the pathogenic mechanism of SARS-CoV-2 ORF8. In addition, with the emergence of two major mutations of ORF8, namely, ORF8L and ORF8S, their differences in antagonizing IFN-I signaling prompted us to study the mechanism by which ORF8L and ORF8S proteins facilitate the immune evasion. We first constructed eukaryotic expression vectors pEGFP-ORF8L and pEGFP-ORF8S, and examined the effect of ORF8L and ORF8S proteins on the activation of the RLR signaling pathway. The results demonstrated that both ORF8L and ORF8S proteins with no difference reduced the mRNA expression of IFN-β, MDA5, RIG-I, ISG15, ISG56, IRF3, and other IFN-related genes induced by poly(I:C) in HeLa cells, indicating that SARS-CoV-2 ORF8 influenced the RIG-I/MDA-5-MAVS signaling-mediated cytosolic dsRNA-sensing pathway and thus inhibited downstream IFN-I production. Moreover, with the stimulation of poly(I:C), ORF8L and ORF8S proteins were also found to inhibit nuclear translocation of IRF3, compared to the cells transfected with empty vectors. Thus, SARS-CoV-2 ORF8L and ORF8S proteins exert an antagonistic effect on RLR pathway by decreasing the translocation of IRF3 into the nucleus, which coincided with previous studies (Rashid et al., 2021). However, the relationship between ORF8 and IRF3 as well as how they function to suppress the host’s innate immunity remain unknown and need to be further studied.

In order to investigate how SARS-CoV-2 ORF8 proteins and IRF3 act together to inhibit the IFN-I pathway, we next examined the possible interacting proteins with ORF8 and determined the relationship between IRF3 and interacting proteins. Previous research has reported that SARS-CoV ORF8b and ORF8ab proteins showed interaction with IRF3 to induce the degradation of IRF3 and thus suppress IFN-β production (Wong et al., 2018), while in SARS-CoV-2, ORF8 induced deamidation of IRF3 and thus inhibited IFN induction by activating CTPS1 (Rao et al., 2021), which suggested that CTPS1 is an important mediator linking ORF8 proteins to IRF3. However, our studies elaborated that both SARS-CoV-2 ORF8L and ORF8S have a significant interaction with HSP90B1. HSP90B1, a member of the heat shock protein 90 (HSP90) family (Hoter et al., 2018), is a molecular chaperone that functions in the processing and trafficking of secreted proteins, as well as in endoplasmic reticulum-associated degradation (ERAD) (Marzec et al., 2012). Furthermore, the gene expression of IFN-β, ISG56, IRF3, and IL-1β induced by poly(I:C) was suppressed when inhibitors were used to downregulate HSP90 expression. Among all of them, IFN-β, ISG56, and IL-1β showed an inhibitory effect of the IFN-I pathway on both ORF8L and ORF8S, but a similar inhibitory trend was not observed in IRF3 with the treatment of HSP90 inhibitors. HSP90B1, also known as GRP94, shares 50% identity to the HSP90 family in cell lines, but the role of HSP90B1 as the major calcium-binding protein in the ER, but only assists specific proteins, would make it distinctly different from other homologs (Marzec et al., 2012); therefore the basic functions of HSP90B1 may vary with that performed by the HSP90 family. Subsequently, we explored the effect of HSP90B1 on the activation of IFN-I pathway factors by silencing HSP90B1 with siRNA. The results demonstrated that with the activation of the IFN-I pathway induced by poly(I:C), IFN-β and IRF3 were significantly downregulated by silencing HSP90B1, which proved that HSP90B1 had a facilitative effect on the IFN-I response. Above all, we speculated that after SARS-CoV-2 ORF8 interacts with HSP90B1, it inhibits the normal expression or proper function of HSP90B1, thereby antagonizing the effective response of the IFN-I pathway. However, the inhibitory effect of ORF8 on HSP90B1 is still unclear, and its specific mechanism, such as the functional domains of HSP90B1, needs further experimental validation.

Although SARS-CoV-2 ORF8 proteins have been proven to inhibit IFN-I signaling pathway and thus predicted to prevent immune clearance and promote viral infection, the ectopic expression of a viral protein may be distinct from that in live coronavirus infection intracellularly or *in vivo* in terms of biological functions. Since ORF8 proteins are accessory proteins and essential for viral functionality, the deletion of the ORF8 protein will have a large impact on the capability of virus infection. The patients infected with the Δ382 variant (382-nucleotide deletion in the ORF8 region of the genome) had lower concentrations of proinflammatory cytokines, chemokines, and growth factors that are strongly associated with severe COVID-19. In addition, Δ382 variants might also be less effective at establishing infection in a new host because of the loss of the immune evasion functions of ORF8 (Young et al., 2020). However, this mutant or ORF8-null SARS-CoV-2 strain is currently not available in our laboratory. Identification of the ORF8 protein mutation that results in attenuation or deficiency of IFN-I pathway inhibition will be more convincing on the function of the ORF8 protein and merits further investigation. Furthermore, animal experiments have not been performed to determine the correlation between infection with the ORF8 mutation strain and the loss of IFN-I response suppression *in vivo*, although the Δ382 variant in patients was demonstrated to disable the immune evasion of SARS-CoV-2. Taken together, experiments in live virus and animal models are needed to improve the understanding of the mechanism by which ORF8 proteins function as an IFN-I antagonist both *in vitro* and *in vivo*, providing guidelines for the development of new antiviral drugs or vaccines targeting ORF8 proteins in SARS-CoV-2.

## Data Availability Statement

The original contributions presented in the study are included in the article/**Supplementary Material** Further inquiries can be directed to the corresponding authors.

## Author Contributions

Conceptualization: BZ and WZ. Data curation: JC, ZL, and XY. Formal analysis: JC and YZ. Funding acquisition: BZ and WZ. Investigation: JC, ZL and XY. Methodology, Project administration, Supervision: BZ. Validation: YZ and JG. Writing—original draft: JC. Writing—review and editing: JC, SZ, SH, JC, JY and BZ. All authors contributed to the article and approved the submitted version.

## Funding

This study was funded by the National Natural Science Foundation (No. 31670168), the Guangdong Provincial Science and Technology (No. 2018B020207006), and the Guangdong Science and Technology Program key projects (No. 2021B1212030014).

## Conflict of Interest

The authors declare that the research was conducted in the absence of any commercial or financial relationships that could be construed as a potential conflict of interest.

## Publisher’s Note

All claims expressed in this article are solely those of the authors and do not necessarily represent those of their affiliated organizations, or those of the publisher, the editors and the reviewers. Any product that may be evaluated in this article, or claim that may be made by its manufacturer, is not guaranteed or endorsed by the publisher.
