# Supplementary material for: Severe Acute Respiratory Syndrome Coronavirus 2 ORF8 Protein Inhibits Type I Interferon Production by Targeting HSP90B1 Signaling
Source: Front Cell Infect Microbiol. 2022 May 23;12:899546. doi: 10.3389/fcimb.2022.899546 (PMC9168264; doi:10.3389/fcimb.2022.899546)
Supplement: Supplementary file 1 [file DataSheet_1.docx]

**Supplementary Table 1**. Primers for Site-directed mutagenesis of target gene ORF8. The Myc-tag sequence was added to 3’ end of ORF8 gene to construct plasmids pEGFP-ORF8L-Myc and pEGFP-ORF8S-Myc.

| Names | Sequence |
| --- | --- |
| ORF8L F primer | 5’-ACTCATCTCTGAAGAGGATCTGggtggcggtgggtctCGggat  ccaccggtcgcc-3’ |
| ORF8L R primer | 5’-ATCCTCTTCAGAGATGAGTTTCTGCTCGATGAAATCTAAAACAA  CACGAACGTCATGAT-3’ |
| ORF8S F primer | 5’-ACTCATCTCTGAAGAGGATCTGggtggcggtgggtctCGggat  ccaccggtcgcc-3’ |
| ORF8S R primer | 5’-ATCCTCTTCAGAGATGAGTTTCTGCTCGATGAAATCTAAAACAA  CACGAACGTCATGAT-3’ |

**Supplementary Table 2**. Primers used for RT-PCR analysis.

| Names | Sequence |
| --- | --- |
| GAPDH F primer | 5’-CATCCTGGGCTACACTGAGC-3’ |
| GAPDH R primer | 5’-AAAGTGGTCGTTGAGGGCAA-3’ |
| IFN-β F primer | 5’- CGCCGCATTGACCATCTA -3’ |
| IFN-β R primer | 5’- GACATTAGCCAGGAGGTTCT -3’ |
| RIG-I F primer | 5’-ATCCCAGTGTATGAACAGCAG-3’ |
| RIG-I R primer | 5’-GCCTGTAACTCTATACCCATGTC-3’ |
| MDA5 F primer | 5’-TGGTCTCGTCACCAATGAAA-3’ |
| MDA5 R primer | 5’-CTCCTGAACCACTGTGAGCA-3’ |
| MAVS F primer | 5'-TTCTAATGCGCTCACCAATCC-3' |
| MAVS R primer | 5'-CATGCTAGTAGGCACTTTGGAGG-3 |
| TBK1 F primer | 5’-TGCACCCTGATATGTATGAGAGA-3’ |
| TBK1 R primer | 5’-AAATGGCAGTGATCCAGTAGC-3’ |
| IKK-γ F primer | 5’-AAGAGCCAACTGTGTGAGATG-3’ |
| IKK-γ R primer | 5’-TTCGCCCAGTACGTCCTGA-3’ |
| IRF3 F primer | 5’-CGGAAAGAAGTGTTGCGGTTAG-3’ |
| IRF3 R primer | 5’-TTTGCCATTGGTGTCAGGAGAG-3’ |
| IRF9 F primer | 5’‐TCATCTGTAAGGGACTAGGAAA-3’ |
| IRF9 R primer | 5’‐AGGTCAGGGAAGAGGGAA-3’ |
| ISG15 F primer | 5′-TGGTGGACAAATCGCACGAA-3′ |
| ISG15 R primer | 5′-CAGGCGCAGATTCATGAAC-3′ |
| ISG56 F primer | 5′-TCTCAGAGGAGCCTGGCTAAG-3′ |
| ISG56 R primer | 5′-CCACACTGTATTTGGTGTCTAGG-3′ |
| CCL5 F primer | 5’-CCGAAAGAACCGCCAAGTGT-3’ |
| CCL5 R primer | 5’-CCCAAGCTAGGACAAGAGCAA-3’ |
| IFITM1 F primer | 5'-CCCCAAAGCCAGAAGATGC-3' |
| IFITM1 R primer | 5'-TCACGGAGTAGGCGAATGCT-3' |
| NF-κB F primer | 5'-AACAGAGAGGATTTCGTTTCCG-3' |
| NF-κB R primer | 5'-TTTGACCTGAGGGTAAGACTTCT-3' |
| IL-1β F primer | 5'-ATGATGGCTTATTACAGTGGCAA-3' |
| IL-1β R primer | 5'-GTCGGAGATTCGTAGCTGGA-3' |
| TNF-α F primer | 5'-CTGGGGCCTACAGCTTTGAT-3' |
| TNF-α R primer | 5'-GGCCTAAGGTCCACTTGTGT-3' |

**Supplementary Table 3**. Mass spectrometry analysis of proteins that may interact with ORF8

| Protein names | Mol. weight [kDa] | Score | Location |
| --- | --- | --- | --- |
| HSPD1 | 61.187 | 175 | Mitochondria |
| RBM8A | 19.934 | 49 | Nucleus, Cytoplasm |
| HSP90B1 | 92.696 | 48 | Endoplasmic reticulum |
| HNRNPA2B1 | 37.464 | 45 | Nucleus, extracellular, cytoplasm |
| CLEC20A | 27 | 44.796 | none |
| VPS16 | 27 | 95.660 | Lysosomes, endosomes |
| NAE1 | 27 | 60.665 | Membrane |
| WDR55 | 26 | 42.557 | Nucleus |
| HSPG2 | 25 | 479.253 | Basement membrane |
| ENO1 | 24 | 47.481 | Cytosol, Cytoplasm |
| HSPA5 | 24 | 72.402 | Endoplasmic reticulum lumen |
| PAX7 | 22 | 55.427 | Nucleus |
| HSPA9 | 19 | 73.920 | Nucleolus, Mitochondria |
| GLYATL1 | 19 | 35.420 | Mitochondria |


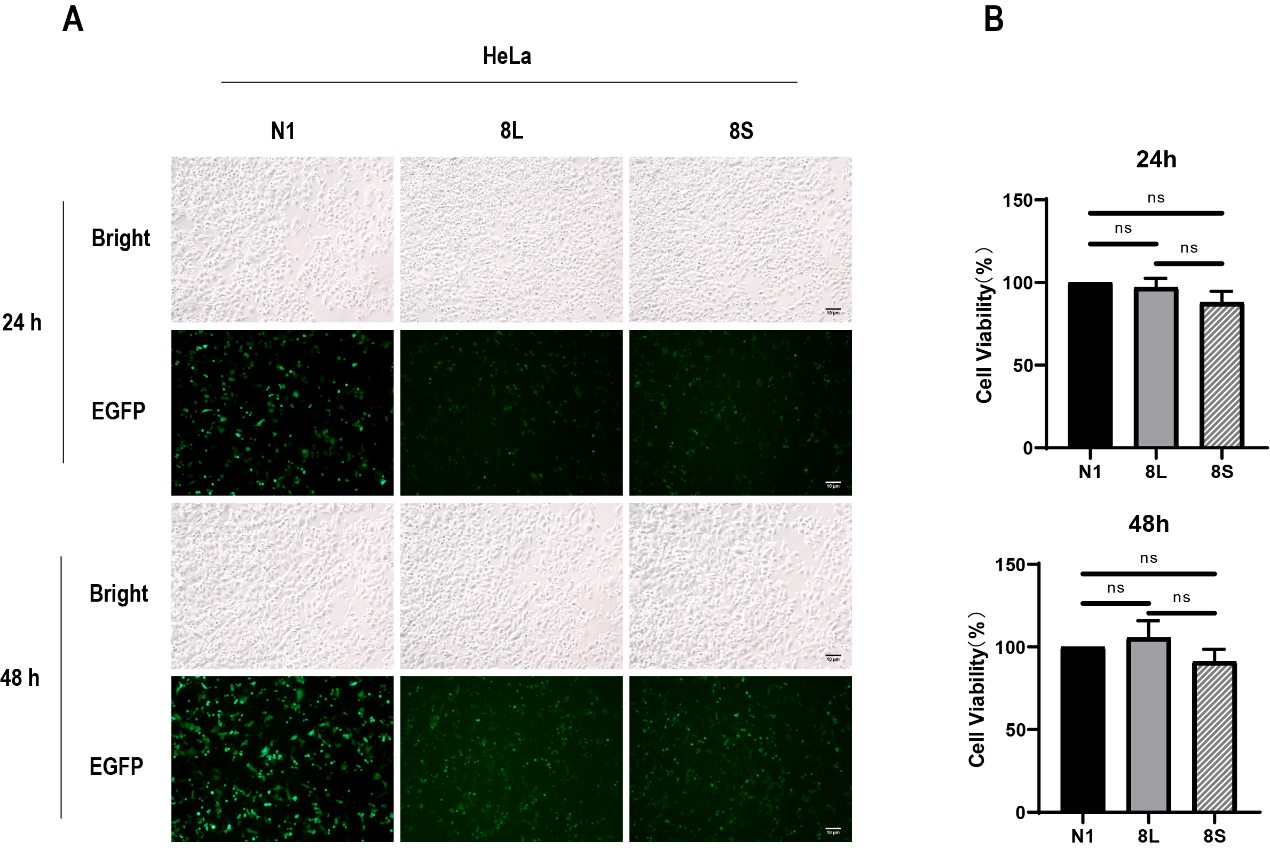


**Supplementary Figure 1**. SARS-CoV-2 ORF8 proteins have no significant impact on the viability of HeLa cells. HeLa cells cultured in 24-well plates were transfected with control vector pEGFP-N1, pEGFP-ORF8L and pEGFP-ORF8S, respectively. At 24 h and 48 h post-transfection, cells were photographed under a fluorescence microscope, using both light and fluorescence (A). Cell viability examined by Cell Counting Kit-8 (CCK8) assays and expressed as the percentage of the control (N1) (taken to be 100%) (B). ns, no significance; *, P < 0.05; **, P < 0.01; *** P < 0.001 versus empty vector (One-way ANOVA). Three independent biological replicates were analyzed, the results of one representative experiment are shown, and the error bars indicate the SD value. Scale bar, 10 μm. pEGFP-N1, N1; pEGFP-ORF8L, 8L; pEGFP-ORF8S, 8S


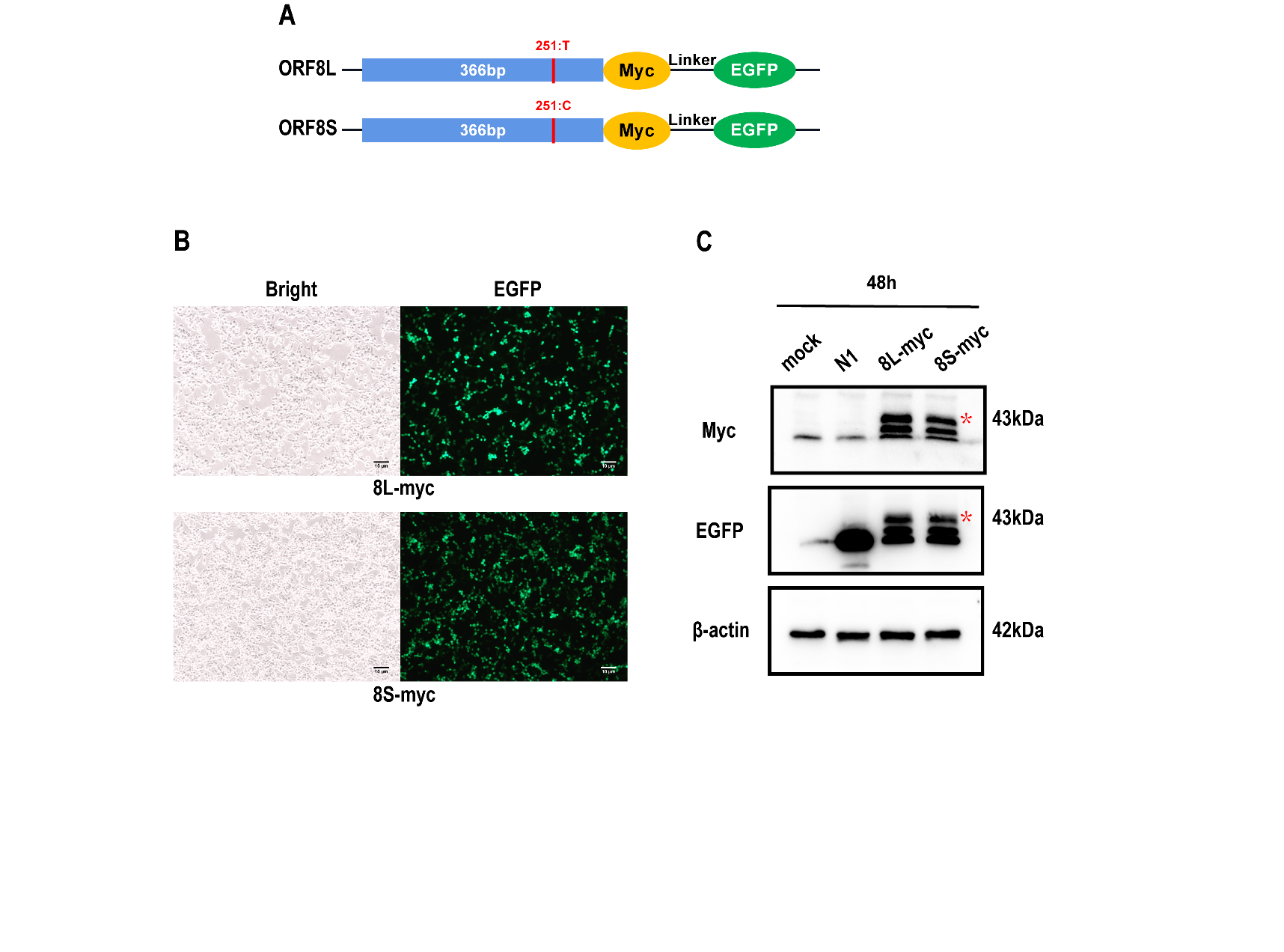


**Supplementary Figure 2**. Expression of pEGFP-ORF8L-Myc and pEGFP-ORF8S-Myc in HEK293T cells. (A) Schematic diagram of ORF8L-Myc and ORF8S-Myc expression vectors. (B) Fluorescence images of 8L-Myc and 8S-Myc proteins. Scale bar, 10 μm. (C) Western blot assay of ORF8L-Myc and ORF8S-Myc proteins. The 8L-Myc and 8S-Myc proteins have a putative molecular weight of nearly 43 kDa, and EGFP protein alone has a molecular weight of 27 kDa. pEGFP-ORF8L-Myc, 8L-Myc; pEGFP-ORF8S-Myc, 8S-Myc
